# The Association between Change in Lifestyle Behaviors and Mental Health Indicators in Immunosuppressed Individuals during the COVID-19 Pandemic

**DOI:** 10.3390/ijerph20032099

**Published:** 2023-01-23

**Authors:** Tara Zeitoun, Audrey Plante, Catherine M. Sabiston, Mélanie Dieudé, Isabelle Doré

**Affiliations:** 1 Department of Nutritional Sciences, Temerty Faculty of Medicine, University of Toronto, Toronto, ON M5S 1A8, Canada; 2 CHUM Research Centre, Montréal, QC H2X 0A9, Canada; 3 Faculty of Kinesiology and Physical Education, University of Toronto, Toronto, ON M5S 2W6, Canada; 4 Department of Microbiology, Infectiology and Immunology, Faculty of medicine, Université de Montréal, Montréal, QC H3C 3J7, Canada; 5 Canadian Donation and Transplant Research Program (CDTRP), Edmonton, AB T6G 2E1, Canada; 6 Héma-Québec, Montréal, QC H4R 2W7, Canada; 7 School of Kinesiology and Physical Activity Sciences, Faculty of Medicine, Université de Montréal, Montréal, QC H3T 1J4, Canada; 8 Social and Preventive Medicine Department, School of Public Health, Université de Montréal, Montréal, QC H3N 1X9, Canada

**Keywords:** COVID-19 pandemic, mental health, physical activity, sleep duration, sedentary behavior, immunosuppressed population

## Abstract

Little is known on how changes in lifestyle behaviors affect mental health among immunosuppressed individuals who observed stricter physical and social distancing measures due to higher risk of complications during the COVID-19 pandemic. This study examines the association between changes in moderate-to-vigorous physical activity (MVPA), sedentary time (ST) and sleep duration following COVID-19 outbreak on mental health indicators of immunosuppressed individuals and their relatives. Participants (*n* = 132) completed an online questionnaire between May and August 2020. Linear regressions were conducted to assess the associations between an increase or decrease in lifestyle behaviors and mental health indicators. Individuals with decreased MVPA and increased ST experienced higher distress, anxiety and depressive symptoms. Those who reported an increase or decrease in sleep had higher levels of stress, distress and depressive symptoms. Decreases in sleep was associated with higher anxiety symptoms. Lifestyle behaviors in the context of a stressful life event such as the COVID-19 pandemic may impact mental health indicators of immunosuppressed individuals and their relatives.

## 1. Introduction

A healthy lifestyle includes a range of activities to encourage habits that reduce the risk of morbidity and increase life expectancy. Such a healthy lifestyle is encompassed by a plethora of habits such as, but not limited to, healthy eating, socialization, regular physical activity and adequate sleep [1]. Regular physical activity is associated with many benefits for physical and psychological health [2,3]. Physical activity is a tool shown to be effective in reducing anxiety [4,5] and depressive symptoms [4], stress [6] and fatigue [7,8], not only in the general population, but also in immunosuppressed individuals [9,10]. Unhealthy changes in lifestyle behaviors (e.g., decreased physical activity, increased sedentary behavior, reduction of quality and quantity of sleep) related to stressful life events could increase stress and anxiety [11,12,13]. As such, encouraging healthy lifestyles may be an effective way to better protect mental health and promote resilience when individuals are faced with stressful life events. 

The instigation of physical and social distancing (PSD) measures related to the COVID-19 pandemic [14] has extensively impacted the mental health of most individuals worldwide [15]. COVID-19 PSD related measures have also posed an obstacle to maintaining healthy lifestyles due to closures of physical activity spaces (e.g., sports centers, recreation facilities, national parks, playgrounds), quarantining for those that may have been in contact with COVID-19, mandatory stay-at-home policies [12,16] and fear of going outside the house for some individuals. The immunosuppressed population’s mental health and lifestyle behaviors are likely to be disproportionately impacted by the PSD measures related to the COVD-19 pandemic. Indeed, immunocompromised individuals are at an increased risk of severe complications due to COVID-19 compared to the general population [17,18], which may make them feel more vulnerable, worried and isolated. Individuals who are immunocompromised are more likely to contract illnesses or be ill for extended periods of times due to their weakened immune system. To protect their loved ones, immunosuppressed patients’ close relatives (parents, guardians, children, siblings, partners) also tend to adopt stricter PSD measures. The physical, mental and social well-being of home care providers in the context of the pandemic has been largely overlooked; specific resources and interventions for people with chronic conditions in the home context need to be considered to support informal home care providers [19,20,21]. Consequently, immunosuppressed individuals and their relatives are a high-risk group in terms of mental health in the current COVID-19 context [22].

In this framework, maintaining healthy lifestyles become a major challenge, which translates to reduced physical activity, increased sedentary behaviors and poor sleep quality [11,23,24,25]. Benefits of physical activity on mental health have been examined in the scope of stressful life events such as the COVID-19 pandemic [16,26]. Results from studies in Canada and other countries with similar PSD measures found that individuals who were more physically active had better mental health [11,12]. A Canadian study revealed that adult participants engage in physical activity as a coping strategy for mental health due to COVID-19 [27]. Alternatively, individuals who decreased their physical activity reported higher feelings of stress, loneliness and depressive symptoms [11] and those who were inactive had higher anxiety levels compared to those who were active [12]. With decreased physical activity and compulsory confinement, sedentary behaviors (e.g., recreational screen time, active screen time b when working, lack of commuting) were observed to increase during the COVID-19 pandemic [28,29]. Recent studies suggest that increased sedentary time is also associated with more depressive or anxiety symptoms [13]. 

Sleep duration is another lifestyle behavior that is vital to mental health and disturbances in sleep quality effect mental and physical performance. Lack of sleep can impair psychological functioning and decision making, jeopardize immune response, increase accidents, lead to mood changes and increase medical expenditures [30]. Moreover, insufficient sleep and poor sleep quality are associated with more depressive and/or anxiety symptoms [31]. Though the relationship between lifestyle factors such as physical activity, sedentary time and sleep time and overall mental health is understood, the effects of a drastic change in lifestyle, as seen in the COVID-19 pandemic, on mental health in vulnerable groups remains equivocal. 

It is unknown whether the benefits of physical activity on mental health observed during COVID-19 in the general population extend to immunosuppressed individuals and their relatives. As this population is likely to adopt strict PSD measures due to their immunosuppression status, maintaining physical activity can represent an even greater challenge compared to the general population. Considering the high level of stress immunosuppressed individuals experience while avoiding illness, it is crucial to assess mental health in a vulnerable population. Examining the relationship between rapid lifestyle changes and mental health outcomes in an immunosuppressed population may help prepare and prevent unwanted mental health outcomes during other similar stressful lifestyle events. 

This study aims to comprehensively investigate the association between changes in lifestyle behaviors and mental health indicators in an immunosuppressed population and their relatives in the context of COVID-19 related stressful life events. We specifically aimed to assess the association between changes in moderate-to-vigorous physical activity (MVPA), sedentary time and sleep duration before and following COVID-19 outbreak on mental health indicators of stress, distress, resilience, anxiety and depressive symptoms. 

## 2. Methods 

### 2.1. Study Design

Our team conducted the COVID-Immuno Study, a cross-sectional study aimed to assess the impact of the COVID-19 pandemic PSD measures on lifestyle behaviors and mental health in an immunosuppressed population and their relatives. Data were collected by our research team from online self-reported questionnaires between May and August 2020. The study received ethics approval from the Ethics Research Committee of the Centre de Recherche du Centre Hospitalier de l’Université de Montréal. 

### 2.2. Data Collection

Participants were recruited through partner organization websites or social media (Canadian Donation and Transplantation Research Program (CDTRP), Kidney Foundation of Canada (KFC), Canadian Transplant Association (CTA) and Cystic Fibrosis Canada (CFC)). The inclusion criteria included: (i) age 15 or older; (ii) English or French-speaking; (iii) being transplanted and immunosuppressed, being immunosuppressed for other reasons, being a close relative (spouse, child, or parent) to an immunosuppressed individual or a donor (iv) having access to the internet. In specific, participants were asked to identify if they were either, (i) a transplant, tissue, or stem cell recipient; (ii) a family member/relative of a transplant/tissue/stem cell recipient or donor; (iii) an organ, tissue, or stem cell donor; (iv) none of the above; or, (v) prefer not to answer. Individuals who selected “none of the above” had an option of explaining if they were immunosuppressed for other reasons, but not transplanted. All eligible participants were invited to complete an online consent form and questionnaire in English or French. 

### 2.3. Sociodemographic Variables

Participants were asked to provide socio-demographic information, including age and employment status and clinical information such as COVID diagnosis, chronic physical health conditions (e.g., osteoarthritis, diabetes, high blood pressure), transplant status and medication usage. 

### 2.4. Mental Health Indicators

Stress levels were captured using a question frequently used in various large surveys such as the Canadian Community Health Survey (CCHS) [32]. Participants were asked to report on a 5-point scale ranging from 1 (not stressful at all) to 5 (extremely stressful) of how stressful most of their days were before and after the COVID-19 pandemic. 

Distress was assessed by asking participants to select on a scale of 0 to 100 (0 = “No distress”; 100 = “Extreme distress”) how much distress they had been experiencing over the past week. Similar Subjective Units of Distress Scale (SUDS) have been used extensively to measure the perceived intensity of distress [33].

Anxiety symptoms were measured using the Generalized Anxiety Disorder 7-item scale (GAD-7) [34]. Participants were asked how often, during the last week, they were bothered by each symptom (e.g., feeling nervous, being restless) on a 4-point scale (0 = not at all, 4 = nearly every day), with higher scores reflecting a more frequent experience of anxiety emotions. Internal consistency [35] of the scores in this sample was 0.89. Evidence of construct validity among the general population has been reported previously [36]. This questionnaire has also been reported to be a suitable screening instrument in more vulnerable settings such as pregnancy and with individuals in primary health care clinics [37,38]. 

Depressive symptoms were measured using the Depressive Symptoms Scale, a 9-item scale based on the Patient Health Questionnaire-9 (PHQ-9) [39]. Participants were asked how often they were bothered by nine specific emotions (e.g., feeling down, feeling tired) relevant for assessing depression over the past week on a 4-point scale (0 = not at all, 4 = nearly every day), with higher scores reflecting a higher frequency of experiencing depressive symptoms. Internal consistency [35] of the scores in this sample was 0.85. Evidence of construct validity has been reported previously in vulnerable populations and meta-analyses [40,41]. 

Resilience, defined as the ability to recover from stress or to bounce back, was captured using the 6-item Brief Resilience Scale (BRS) [42]. Participants were asked to report on a 5-point scale (1 = strongly disagree, 5 = strongly agree) how much they agree or disagree with statements related to resilience (e.g., I usually come through difficult times with little trouble), with higher scores reflecting a stronger frequency of feeling resilient. Internal consistency [35] of the scores in this sample was 0.85. Evidence of construct validity has been reported previously [43,44]. 

### 2.5. Lifestyle Behaviors

Informed by the International Physical Activity Questionnaire (IPAQ) [45] and other valid and reliable measures of physical activity [46], items were developed for the current study. Participants self-reported days of MVPA for two specific periods, before COVID-19 and in the past 7 days. To help participants define the inception of the COVID-19 pandemic, the following information was provided: “The next questions asks about your physical activity, sedentary time and sleep. For each question, we will ask you to think about a typical week before the COVID-19* pandemic and the past 7 days (*COVID-19 was declared a global pandemic on 11 March 2020)”.

**Moderate-to-vigorous physical activity** (MVPA) was measured by asking participants, “How many days per week did you do vigorous aerobic activity, such as aerobics or fast bicycling?”, and “How many days per week did you do moderate aerobic activity, such as jogging, bicycling at a regular pace, or doubles tennis?”, respectively. They were asked to report each activity frequency for two reference periods: before COVID-19 and over the past 7 days. The average number of days spent in moderate-vigorous physical activity was calculated for both before COVID-19 and for the last 7 days by summing the average number of days spent at each of the two intensities. Change in physical activity was categorized nominally as decreased whenever a decline of at least one day in the average number of days in MVPA was observed, as stable when no change was observed and as increased when an increase of at least one day was observed. We utilized one day as the unit of change as this was the smallest unit in the response option. 

**Sedentary time** was assessed similarly to MVPA. Participants were asked to report the average number of hours and minutes spent in sedentary positions (e.g., sitting, lying down) on a typical weekday and on a weekend day on a typical week before COVID-19 and in the past seven days, respectively. Sedentary time averages for a typical week prior to COVID-19 and for the last seven days were calculated with the following formula: ((no. of minutes of sedentary time on weekdays) × 5 + (no. of minutes of sedentary time on weekend days) × 2)/7. We categorized individuals nominally by their pre- and post-COVID-19 change in sedentary time into either decreased (decrease of ≥10 min), increased (increase of ≥10 min) or stable (decrease or increased of <10 min).For measurement, 10-minute bouts of change for sedentary time were included to capture perceived change, based on previous literature assessing changes in MVPA bouts [47]. 

**Sleep duration** before and after the onset of COVID-19 were measured by asking the participants to report the average amount of hours and minutes slept on a typical weekday and on a weekend day in a typical week before COVID-19 and in the past seven days, respectively. Sleep time averages for a typical week prior to COVID-19 and for the last seven days were calculated with the following formula: ((no. of minutes sleeping on weeknights) × 5 + (no. of minutes sleeping on weekend nights) × 2)/7. We categorized individuals nominally by their pre- and post-COVID-19 change in sleep duration to either decreased (decrease of ≥10 min), increased (increase of ≥10 min) or stable (decrease or increase of <10 min) and 10-minute bouts of change for sleep duration were included to capture perceived change, based on previous literature assessing changes in MVPA bouts [47].

### 2.6. Statistical Analyses

Preliminary analyses included descriptive analyses to assess distributions, identify outliers, compute proportions, means and standard deviations. Descriptive analyses were also conducted to compare each mental health indicator (stress, distress, resilience, anxiety, and depression symptoms) according to lifestyle behavior (moderate-vigorous physical activity, sedentary time and sleep duration over the past 7 days) and change categories (increase, decrease, stable). 

Multivariate linear regressions were used to assess whether an increase or decrease in lifestyle behaviors MVPA, sedentary time and sleep duration is associated with each mental health indicator, for stress, distress, resilience, anxiety, and depressive symptoms, compared to no changes. Linear regressions were chosen to estimate the association between the categorical exposure of lifestyle behavior changes and continuous outcome of mental health indicators. Separate models were conducted for each lifestyle behavior with each specific mental health indicator. Models were adjusted for potential confounders identified in the literature, which include age [48], sex [49,50] and physical diagnosis [51,52]. All covariates were determined a priori and chosen based on theoretical grounds and known associations with the exposure and outcome of interest. All analysis was conducted using R (version 4.2.0) [53] and the “rms” package (version 6.1) [54].

## 3. Results

Participants’ characteristics are presented in Table 1. After excluding individuals with missing data (*n* = 5), 132 participants (65% females) remained in the analytical sample. The majority of the participants were aged between 35 and 69 years old (77%). Most of the participants (76%) were either a transplant, tissue, or stem cell recipient, 18% of the participants were relatives of a transplant/tissue/stem donor, 3% of the population were organ, tissue or stem cell donors and 3% were immunosuppressed for other undisclosed reasons. There were no significant differences in the reporting of lifestyle behaviors, sedentary time, sleep duration and MVPA, between participant groups. A majority of participants were not on anxiolytic or anti-depressant medications (80%). Participants reported decreasing their MVPA by an average of 1 day/week (SD = 3.1 days/week) and increasing their sedentary time by an average of 106.8 min per day (SD = 131.6 min/day) since the onset of the COVID-19 pandemic. Participants reported, on average, a minimal decrease in sleep time (mean (SD) = −1.4 min/day (SD = 80.7 min/day)). When grouped in lifestyle behaviors change categories, we found that 48% of participants reported stable MVPA, followed by 33% who decreased their MVPA and 19% who reported increasing their MVPA. Most of the participants reported an increased in sedentary time (68%), while only 9% decreased and 23% remained stable. In our sample, 39% increased their sleep duration, 33% decreased and 28% remained stable. 

Means and standard deviations for each mental health outcome according to lifestyle behavior change groups are reported in Table 2. These descriptive statistics suggest that participants who decrease MVPA report higher level of stress, distress anxiety and depressive symptoms compared to those who remain stable or increase MVPA. Participants who increase sedentary time report higher distress, anxiety and depressive symptoms compared to those who remain stable or decrease sedentary time. For sleep, participants who remain stable report lower stress level, distress, anxiety and depressive symptoms compared to those who report changes in their sleeping habits, whether these increased or decreased. Detailed measures for lifestyle behavior (MVPA, sleep duration and sedentary time) before COVID and in the past 7 days, and different means and standard deviations were extracted per change category (decrease, stable, increase) and are presented in Supplementary Table S1. 

Results from the linear regressions (Table 3) show that, compared to participants for whom MVPA remain stable, participants who decreased MVPA report higher distress ($\hat{\beta }$(95% CI) = 9.09 (0.52, 17.65)), anxiety symptoms ($\hat{\beta }$(95% CI) = 1.89 (0.18, 3.61)) and depressive symptoms ($\hat{\beta }$ (95% CI) = 1.97 (0.007, 3.88)). An increase in sedentary time was associated with higher stress ($\hat{\beta }$(95% CI) = 0.44 (0.05, 0.83)), distress ($\hat{\beta }$(95% CI) = 14.15 (4.16, 24.15)), anxiety symptoms ($\hat{\beta }$(95% CI) = 2.29 (0.20, 4.38)) and depressive symptoms ($\hat{\beta }$(95% CI) = 2.65 (0.35, 4.95)) compared to stable sedentary time. A decrease in sedentary time was associated with higher stress (β(95% CI) = 0.32 (0.26, 0.92)) compared to stable sedentary time. Compared to participants who report no change in sleep duration, participants reporting increased sleep duration present higher stress ($\hat{\beta }$(95% CI) = 0.55 (0.18, 0.91)), distress ($\hat{\beta }$(95% CI) = 13.09 (3.34, 22.84)) and depressive symptoms ($\hat{\beta }$ (95% CI) = 2.53 (0.35, 4.72)). Similarly, a decrease in sleep duration was associated with higher stress ($\hat{\beta }$(95% CI) = 0.72 (0.33, 1.11)), distress ($\hat{\beta }$(95% CI) = 13.58 (3.31, 23.85)), anxiety symptoms ($\hat{\beta }$(95% CI) = 2.29 (0.20, 4.38)) and depressive symptoms ($\hat{\beta }$(95% CI) = 2.65 (0.35, 4.95)) compared to those reporting no change in sleep duration. Changes in MVPA, sedentary time or sleep duration, whether it increased or decreased, were not associated with resilience. 

Sensitivity analyses were conducted, assessing only those who were immunocompromised (*n* = 108), whereby all the same associations were observed between changes in lifestyle behaviors and mental health indictors, as seen in Supplementary Table S2. The only difference observed was that an increase in sleep duration was positively associated with anxiety symptoms ($\hat{\beta }$(95% CI) = 2.43 (0.09, 4.79)), whereby in the main analyses, this finding was not significant ($\hat{\beta }$(95% CI) = 1.70 (−0.28, 3.69)). These sensitivity analyses depict that after excluding the relatives of participants, changes in lifestyle behaviors are still associated with mental health indicators. 

## 4. Discussion 

This study aimed to assess the association between changes in the lifestyle behaviors of MVPA, sedentary time and sleep duration on mental health indicators during the COVID-19 pandemic lockdown among immunosuppressed individuals and their relatives. Our results indicate that unhealthy changes in lifestyle behaviors are associated with poor mental health outcomes. Specifically, decreases in MVPA are associated with higher stress, anxiety and depressive symptoms whereas increased in sedentary time is associated higher stress, distress, anxiety and depressive symptoms. Changes in sleep duration, whether it is an increased or a decrease, were associated with negative mental health outcomes: higher stress, distress and depressive symptoms. A similar trend was observed in our sensitivity analyses, after excluding the relatives of participants. Based on these findings among an understudied subgroup of immunosuppressed individuals, lifestyle behavior changes during stressful life events such as rapid outbreaks of infectious diseases have strong associations with mental health.

Extensive research supports the positive association between physical activity and mental health, even during the COVID-19 pandemic, where decreased physical activity led to worsening of mental health [55,56]. Studies investigating the associations between lifestyle behaviors and mental health suggest that during the compulsory confinements, individuals were less physically active and had more depression, anxiety and stress [11,12,57]. The current results similarly suggest that individuals who decreased their physical activity had higher distress and depressive symptoms. It is likely that physical and social distancing measures imposed a structural barrier to maintaining physical active including transportation and daily errands and this change in behavior was associated with poorer mental health outcomes. 

To support the current findings that sedentary behavior increased and was related to worsened mental health indicators, a recent meta-analysis and systematic review observed worse global mental health, depression, anxiety and quality of life across the age span with increased sedentary time over the COVID-19 pandemic [58,59,60]. Like physical activity decreases, an increase in sedentary behavior during the pandemic was inevitable due to public health confinement requirements. With the known negative associations of sedentary time and mental health [11,61], it was only expected that an increase in sedentary time would negatively influence mental health parameters. 

Our results are in line with previous studies suggesting that the COVID-19 pandemic had negative effects on sleep duration. A systematic review and meta-analysis examining sleep problems during the pandemic found that 35.7% of all populations had more sleep problems during the first eight months of the pandemic [62]. Significant sleep disturbances were reported during the pandemic across different populations such as adolescents and children [63], university students [64] and nurses [65]. To our knowledge, our findings are the first to suggest that not only were there changes in sleep during the pandemic, but that these changes significantly affected mental health status in immunosuppressed populations and their relatives. Resilience was not seen to be associated with any change in lifestyle behavior in our population. Though it has been observed that overall resilience was lower amongst adults in the USA during the COVID-19 pandemic [66,67], our results suggested that resilience was not significantly associated with lifestyle changes. 

There is evidence to suggest that the increased mental health indicators reported among immunosuppressed individuals may be due to an increased fear of contracting COVID-19 and the increased adherence to COVID-19 preventative behaviors during the mandatory stay at home order [68,69]. However, this study provides an important contribution to the limited evidence examining whether lifestyle behaviors change in physical activity, sleep and sedentary behavior relate to mental health among the immunosuppressed population and their relatives. Indeed, healthier lifestyle choices such as increased physical activity, less sedentary time and adequate sleep time are strong indicators of mental health [11,70] and such associations extend to the immunosuppressed population. An individual who is already vulnerable due to their health status may face more stress in anticipation of an increased risk of illness or infection, not only during the pandemic [71,72], but in other time periods of restraint as well. Our results suggest that a healthy lifestyle, by increasing physical activity, decreasing sedentary time and maintaining adequate sleep levels, could help mitigate the negative impact of the numerous stressful events in life that this particularly vulnerable population may already be facing. 

Limitations of this study include the cross-sectional design which precludes inference of causality. Pre-COVID lifestyle behaviors measurements are subject to recall bias which could results in misclassification of change in MVPA, sedentary time and sleep duration. The use of a self-reported questionnaire is also subject to misclassification. Additionally, the use of a convenient sample may limit the generalizability of the findings outside the scope of immunocompromised individuals. Moreover, the 10-min cut-offs used for sedentary behavior and sleep duration may be underestimating change in our population. Though the brief measurement of MVPA has previously been used before [27], the measurement of MVPA as days/week rather than minutes/day poses itself as a limitation as we are unable to detect the time per day spent doing MVPA. Finally, we acknowledge that the estimated associations between change in lifestyle behaviors and mental health indicators could be affected by residual confounding.

## 5. Conclusions

The current findings indicate that the lifestyle behavior changes during a stressful life event, such as the COVID-19 pandemic, were associated with negative mental health indicators in immunosuppressed populations. Assessing such a vulnerable population is crucial considering the stronger confinement requirements for such a population and their risk for health complications in stressful experiences. Future studies should investigate mental health indicators, including positive aspects such as resilience, in transplanted and immunosuppressed populations in various stressful life contexts.

## Figures and Tables

**Table 1:** Participant Characteristics (*n* = 132) ^a^.

Participant Characteristics	*n* (%) or (mean ± SD)
Sex, (female)	86 (65)
Age, *n* (%)	
Under 18 years old	5 (4)
18 to 34 years old	20 (15)
35 to 54 years old	52 (39)
55 to 69 years old	51 (39)
70 years old or over	4 (3)
Transplant Status, *n* (%)	
A transplant, tissue or stem cell recipient	100 (76)
An organ, tissue or stem cell donor	4 (3)
A family member/relative of recipient or donor	24 (18)
Immunosuppressed (other reasons)	4 (3)
Medication Usage, *n* (%)	
No	105 (80)
Yes	26 (20)
Prefer not to answer	1 (1)
Employment, *n* (%)	
Employed full time	41 (31)
Employed part time	13 (10)
Self-employed	9 (7)
Unemployed	7 (5)
Retired	25 (19)
On temporary leave (illness, work related accident, maternity-paternity)	13 (10)
Student	10 (8)
Other	13 (10)
Average change in MVPA, (days/week) ^a^	−1.0 ± 3.1
Change in MVPA, *n* (%)	
Decrease	43 (33)
Stable	63 (48)
Increase	26 (19)
Average change in sedentary time, (min/day) ^a^	106.8 ± 131.6
Change in sedentary time categories, *n* (%)	
Decrease	12 (9)
Stable	30 (23)
Increase	90 (68)
Average change in sleep, (min/day) ^a^	−1.4 ± 80.7
Change in sleep duration categories, *n* (%)	
Decrease	44 (33)
Stable	37 (28)
Increase	51 (39)
Stress, (mean ± SD)	3.4 ± 0.9
Anxiety Symptoms, (mean ± SD)	5.6 ± 4.7
Distress, (mean ± SD)	32.7 ± 23.8
Depressive Symptoms, (mean ± SD)	6.4 ± 5.2
Resilience, (mean ± SD)	21.8 ± 5.0

^a^ Values are unadjusted means ± standard deviations.

**Table 2:** Mental health outcomes according to lifestyle behavior change (*n* = 132) ^a^.

**Moderate to Vigorous Physical Activity**	**Decrease** **(*n* = 43)**	**Stable** **(*n* = 63)**	**Increase** **(*n* = 26)**
Stress	3.5 ± 0.9	3.1 ± 0.9	3.2 ± 1.0
Distress	37.4 ± 24.6	28.9 ± 23.1	29.6 ± 22.3
Resilience Scale	21.4 ± 5.2	22.0 ± 4.7	22.4 ± 5.1
Anxiety Symptoms	6.7 ± 5.3	4.8 ± 4.2	4.9 ± 3.9
Depression Symptoms	7.4 ± 5.6	5.5 ± 4.7	5.6 ± 5.1
**Sedentary Time**	**Decrease** **(*n* = 12)**	**Stable** **(*n* = 30)**	**Increase** **(*n* = 90)**
Stress	3.6 ± 0.9	3.2 ± 0.8	3.5 ± 1.0
Distress	29.2 ± 20.68	22.2 ± 21.3	36.7 ± 24.0
Resilience Scale	21.1 ± 5.74	22.8 ± 5.1	21.6 ± 4.8
Anxiety Symptoms	5.5 ± 3.37	3.6 ± 3.6	6.3 ± 5.0
Depression Symptoms	6.0 ± 4.86	4.3 ± 4.6	7.1 ± 5.3
**Sleep Duration**	**Decrease** **(*n* = 44)**	**Stable** **(*n* = 37)**	**Increase** **(*n* = 51)**
Stress	3.7 ± 0.9	3.0 ± 0.7	3.5 ± 1.1
Distress	38.5 ± 23.9	21.6 ± 18.0	35.8 ± 25.1
Resilience Scale	21.6 ± 4.9	22.8 ± 5.5	21.3 ± 4.7
Anxiety Symptoms	6.8 ± 4.5	3.8 ± 3.6	5.9 ± 5.3
Depression Symptoms	7.5 ± 5.5	4.0 ± 3.8	7.0 ± 5.4

^a^ Values are unadjusted means ± standard deviations.

**Table 3:** Associations between changes in lifestyle behaviors on mental health indicators (*n* = 132).

	Stress			Distress			Resilience			Anxiety Symptoms			Depressive Symptoms		
	$\hat{\beta }$	95% CI	*p* ^a^	$\hat{\beta }$	95% CI	*p* ^a^	$\hat{\beta }$	95% CI	*p* ^a^	$\hat{\beta }$	95% CI	*p* ^a^	$\hat{\beta }$	95% CI	*p* ^a^
MVPA ^a^															
Stable (*n* = 63)	Ref			Ref			Ref			Ref			Ref		
Increase (*n* = 26)	−0.17	0.58, 0.24	0.41	3.14	−7.60, 13.89	0.56	0.29	−2.17, 2.78	0.81	0.54	−1.56, 2.74	0.59	0.41	−1.96, 2.80	0.73
Decrease (*n* = 43)	0.20	0.13, 0.53	0.22	**9.09**	**0.52, 17.65**	**0.04**	−0.62	−2.59, 1.35	0.53	**1.89**	**0.18, 3.61**	**0.03**	**1.97**	**0.07, 3.88**	**0.04**
Sedentary time															
Stable (*n* = 30)	Ref			Ref			Ref			Ref			Ref		
Increase (*n* = 90)	**0.44**	**0.05, 0.83**	**0.03**	**14.15**	**4.16, 24.15**	**0.005**	0.97	−3.30, 1.37	0.41	**2.37**	**0.34, 4.40**	**0.02**	**2.39**	**0.13, 4.65**	**0.03**
Decrease (*n* = 12)	**0.32**	**0.26, 0.92**	**0.02**	1.98	−13.10, 17.06	0.79	−1.44	−4.97, 2.08	0.42	0.66	−2.40, 3.72	0.67	0.49	−2.92, 3.90	0.77
Sleep duration															
Stable (*n* = 37)	Ref			Ref			Ref			Ref			Ref		
Increase (*n* = 51)	**0.55**	**0.18, 0.91**	**0.003**	**13.09**	**3.34, 22.84**	**0.009**	−1.29	−3.57, 0.97	0.26	1.70	−0.28, 3.69	0.09	**2.53**	**0.35, 4.72**	**0.02**
Decrease (*n* = 44)	**0.72**	**0.33, 1.11**	**<0.001**	**13.58**	**3.31, 23.85**	**0.009**	−0.94	−3.33, 1.45	0.44	**2.29**	**0.20, 4.38**	**0.03**	**2.65**	**0.35, 4.95**	**0.02**

^a^ *p* value adjusted for age, sex and physical illness diagnosis. ^b^ MVPA = Moderate-to-vigorous physical activity. Bold indicate statistically significant results at *p* < 0.05.

## Data Availability

The datasets used and/or analyzed during the current study are available from the corresponding author on reasonable request.

## Supplementary Materials

The following supporting information can be downloaded at: https://www.mdpi.com/article/10.3390/ijerph20032099/s1, Table S1. Continuous averages of lifestyle behaviors according to categorical measures; Table S2. Associations between changes in lifestyle behaviors on mental health indicators in the subsample restricted to immunosuppressed individuals (*n* = 108).

## References

[1] Byrne D.W., Rolando L.A., Aliyu M.H., McGown P.W., Connor L.R., Awalt B.M., Holmes M.C., Wang L., Yarbrough M.I. (2016). Modifiable Healthy Lifestyle Behaviors: 10-Year Health Outcomes From a Health Promotion Program. Am. J. Prev. Med..

[2] Czosnek L., Lederman O., Cormie P., Zopf E., Stubbs B., Rosenbaum S. (2019). Health benefits, safety and cost of physical activity interventions for mental health conditions: A meta-review to inform translation efforts. Ment. Health Phys. Act..

[3] Warburton D.E., Bredin S.S. (2017). Health benefits of physical activity: A systematic review of current systematic reviews. Curr. Opin. Cardiol..

[4] Schuch F.B., Vancampfort D., Firth J., Rosenbaum S., Ward P.B., Silva E.S., Hallgren M., Ponce De Leon A., Dunn A.L., Deslandes A.C. (2018). Physical activity and incident depression: A meta-analysis of prospective cohort studies. Am. J. Psychiatr..

[5] Stubbs B., Koyanagi A., Hallgren M., Firth J., Richards J., Schuch F., Rosenbaum S., Mugisha J., Veronese N., Lahti J. (2017). Physical activity and anxiety: A perspective from the World Health Survey. J. Affect. Disord..

[6] Moylan S., Eyre H., Maes M., Baune B., Jacka F., Berk M. (2013). Exercising the worry away: How inflammation, oxidative and nitrogen stress mediates the beneficial effect of physical activity on anxiety disorder symptoms and behaviours. Neurosci. Biobehav. Rev..

[7] Brandenbarg D., Korsten J.H., Berger M.Y., Berendsen A.J. (2018). The effect of physical activity on fatigue among survivors of colorectal cancer: A systematic review and meta-analysis. Support. Care Cancer.

[8] Oberoi S., Robinson P.D., Cataudella D., Culos-Reed S.N., Davis H., Duong N., Gibson F., Götte M., Hinds P., Nijhof S.L. (2018). Physical activity reduces fatigue in patients with cancer and hematopoietic stem cell transplant recipients: A systematic review and meta-analysis of randomized trials. Crit. Rev. Oncol. Hematol..

[9] Segatto B.L., Sabiston C.M., Harvey W.J., Bloom G.A. (2013). Exploring relationships among distress, psychological growth, motivation, and physical activity among transplant recipients. Disabil. Rehabil..

[10] Takahashi A., Hu S.L., Bostom A. (2018). Physical activity in kidney transplant recipients: A review. Am. J. Kidney Dis..

[11] Meyer J., McDowell C., Lansing J., Brower C., Smith L., Tully M., Herring M. (2020). Changes in Physical Activity and Sedentary Behavior in Response to COVID-19 and Their Associations with Mental Health in 3052 US Adults. Int. J. Environ. Res. Public Health.

[12] Lesser I.A., Nienhuis C.P. (2020). The Impact of COVID-19 on Physical Activity Behavior and Well-Being of Canadians. Int. J. Environ. Res. Public Health.

[13] Lee E., Kim Y. (2019). Effect of university students’ sedentary behavior on stress, anxiety, and depression. Perspect. Psychiatr. Care.

[14] WHO Advice for public: Coronavirus diseas (COVID-19). https://www.who.int/emergencies/diseases/novel-coronavirus-2019/advice-for-public#:~:text=Keep%20physical%20distance%20of%20at,and%20in%20poorly%20ventilated%20settings.

[15] Hossain M.M., Tasnim S., Sultana A., Faizah F., Mazumder H., Zou L., McKyer E.L.J., Ahmed H.U., Ma P. (2020). Epidemiology of mental health problems in COVID-19: A review. F1000Research.

[16] Wolf S., Seiffer B., Zeibig J.M., Welkerling J., Brokmeier L., Atrott B., Ehring T., Schuch F.B. (2021). Is Physical Activity Associated with Less Depression and Anxiety During the COVID-19 Pandemic? A Rapid Systematic Review. Sport. Med..

[17] Banerjee D., Popoola J., Shah S., Ster I.C., Quan V., Phanish M. (2020). COVID-19 infection in kidney transplant recipients. Kidney Int..

[18] Bhoori S., Rossi R.E., Citterio D., Mazzaferro V. (2020). COVID-19 in long-term liver transplant patients: Preliminary experience from an Italian transplant centre in Lombardy. Lancet Gastroenterol. Hepatol..

[19] Chan E.Y., Lo E.S., Huang Z., Kim J.H., Hung H., Hung K.K., Wong E.L., Wong S.Y., Gobat N. (2020). Characteristics and well-being of urban informal home care providers during COVID-19 pandemic: A population-based study. BMJ Open.

[20] Whitley E., Reeve K., Benzeval M. (2021). Tracking the mental health of home-carers during the first COVID-19 national lockdown: Evidence from a nationally representative UK survey. Psychol. Med..

[21] Li Q., Zhang H., Zhang M., Li T., Ma W., An C., Chen Y., Liu S., Kuang W., Yu X. (2020). Prevalence and Risk Factors of Anxiety, Depression, and Sleep Problems Among Caregivers of People Living With Neurocognitive Disorders During the COVID-19 Pandemic. Front. Psychiatr..

[22] Al-Rahimi J.S., Nass N.M., Hassoubah S.A., Wazqar D.Y., Alamoudi S.A. (2021). Levels and predictors of fear and health anxiety during the current outbreak of COVID-19 in immunocompromised and chronic disease patients in Saudi Arabia: A cross-sectional correlational study. PLoS ONE.

[23] Castañeda-Babarro A., Arbillaga-Etxarri A., Gutiérrez-Santamaría B., Coca A. (2020). Physical Activity Change during COVID-19 Confinement. Int. J. Environ. Res. Public Health.

[24] Sepúlveda-Loyola W., Rodríguez-Sánchez I., Pérez-Rodríguez P., Ganz F., Torralba R., Oliveira D.V., Rodríguez-Mañas L. (2020). Impact of Social Isolation Due to COVID-19 on Health in Older People: Mental and Physical Effects and Recommendations. J. Nutr. Health Aging.

[25] Morin C.M., Carrier J., Bastien C., Godbout R. (2020). Sleep and circadian rhythm in response to the COVID-19 pandemic. Can. J. Public Health.

[26] Schuch F.B., Bulzing R.A., Meyer J., López-Sánchez G.F., Grabovac I., Willeit P., Vancampfort D., Caperchione C.M., Sadarangani K.P., Werneck A.O. (2021). Moderate to vigorous physical activity and sedentary behavior changes in self-isolating adults during the COVID-19 pandemic in Brazil: A cross-sectional survey exploring correlates. Sport. Sci. Health.

[27] Faulkner G., Rhodes R.E., Vanderloo L.M., Chulak-Bozer T., O’Reilly N., Ferguson L., Spence J.C. (2020). Physical activity as a coping strategy for mental health due to the COVID-19 virus: A potential disconnect among Canadian adults?. Front. Commun..

[28] Romero-Blanco C., Rodríguez-Almagro J., Onieva-Zafra M.D., Parra-Fernández M.L., Prado-Laguna M.D.C., Hernández-Martínez A. (2020). Physical Activity and Sedentary Lifestyle in University Students: Changes during Confinement Due to the COVID-19 Pandemic. Int. J. Environ. Res. Public Health.

[29] Dunton G.F., Do B., Wang S.D. (2020). Early effects of the COVID-19 pandemic on physical activity and sedentary behavior in children living in the U.S. BMC Public Health.

[30] Medic G., Wille M., Hemels M.E. (2017). Short- and long-term health consequences of sleep disruption. Nat. Sci. Sleep.

[31] Nyer M., Farabaugh A., Fehling K., Soskin D., Holt D., Papakostas G.I., Pedrelli P., Fava M., Pisoni A., Vitolo O. (2013). Relationship between sleep disturbance and depression, anxiety, and functioning in college students. Depress. Anxiety.

[32] Government of Canada (2011). Canadian Community Health Survey-Mental Health (CCHS). https://www23.statcan.gc.ca/imdb/p2SV.pl?Function=getSurvey&Id=119789.

[33] McCabe R.E. (2015). Subjective units of distress scale. J. Phobias Psychol. Irrational. Fear.

[34] Spitzer R.L., Kroenke K., Williams J.B., Löwe B. (2006). A brief measure for assessing generalized anxiety disorder: The GAD-7. Arch. Intern. Med..

[35] Cronbach L.J. (1951). Coefficient alpha and the internal structure of tests. Psychometrika.

[36] Löwe B., Decker O., Müller S., Brähler E., Schellberg D., Herzog W., Herzberg P.Y. (2008). Validation and standardization of the Generalized Anxiety Disorder Screener (GAD-7) in the general population. Med. Care.

[37] Sidik S.M., Arroll B., Goodyear-Smith F. (2012). Validation of the GAD-7 (Malay version) among women attending a primary care clinic in Malaysia. J. Prim. Health Care.

[38] Gong Y., Zhou H., Zhang Y., Zhu X., Wang X., Shen B., Xian J., Ding Y. (2021). Validation of the 7-item Generalized Anxiety Disorder scale (GAD-7) as a screening tool for anxiety among pregnant Chinese women. J. Affect. Disord..

[39] Kroenke K., Spitzer R.L., Williams J.B. (2001). The PHQ-9: Validity of a brief depression severity measure. J. Gen. Intern. Med..

[40] Kroenke K., Spitzer R.L., Williams J.B.W., Löwe B. (2010). The Patient Health Questionnaire Somatic, Anxiety, and Depressive Symptom Scales: A systematic review. Gen. Hosp. Psychiatr..

[41] Costantini L., Pasquarella C., Odone A., Colucci M.E., Costanza A., Serafini G., Aguglia A., Belvederi Murri M., Brakoulias V., Amore M. (2021). Screening for depression in primary care with Patient Health Questionnaire-9 (PHQ-9): A systematic review. J. Affect. Disord..

[42] Smith B.W., Dalen J., Wiggins K., Tooley E., Christopher P., Bernard J. (2008). The brief resilience scale: Assessing the ability to bounce back. Int. J. Behav. Med..

[43] Sánchez J., Estrada-Hernández N., Booth J., Pan D. (2021). Factor structure, internal reliability, and construct validity of the Brief Resilience Scale (BRS): A study on persons with serious mental illness living in the community. Psychol. Psychother..

[44] da Silva-Sauer L., de la Torre-Luque A., Smith B.W., Andrade S., Fernández-Calvo B., MCMCL (2021). Brief Resilience Scale (BRS) Portuguese Version: Validity and metrics for the older adult population. Aging Ment. Health.

[45] Craig C.L., Marshall A.L., Sjöström M., Bauman A.E., Booth M.L., Ainsworth B.E., Pratt M., Ekelund U., Yngve A., Sallis J.F. (2003). International physical activity questionnaire: 12-country reliability and validity. Med. Sci. Sport. Exerc..

[46] Godin G. (2011). The Godin-Shephard leisure-time physical activity questionnaire. Health Fit. J. Can..

[47] Brannan M.G.T., Foster C.E., Timpson C.M., Clarke N., Sunyer E., Amlani A., Murphy M.H. (2019). Active 10—A new approach to increase physical activity in inactive people in England. Prog. Cardiovasc. Dis..

[48] Wilson J.M., Lee J., Shook N.J. (2021). COVID-19 worries and mental health: The moderating effect of age. Aging Ment. Health.

[49] Meyer O.L., Castro-Schilo L., Aguilar-Gaxiola S. (2014). Determinants of mental health and self-rated health: A model of socioeconomic status, neighborhood safety, and physical activity. Am. J. Public Health.

[50] Holingue C., Budavari A.C., Rodriguez K.M., Zisman C.R., Windheim G., Fallin M.D. (2020). Sex Differences in the Gut-Brain Axis: Implications for Mental Health. Curr. Psychiatr. Rep..

[51] Smith A.J., Wright H., Griffin B.J., Ehman A.C., Shoji K., Love T.M., Morrow E., Locke A., Call M., Kerig P.K. (2021). Mental health risks differentially associated with immunocompromised status among healthcare workers and family members at the pandemic outset. Brain Behav. Immun. Health.

[52] Kang N.R., Ahn Y.H., Park E., Choi H.J., Kim S.H., Cho H., Cho M.H., Shin J.I., Lee J.H., Park Y.S. (2019). Mental health and psychosocial adjustment in pediatric chronic kidney disease derived from the KNOW-Ped CKD study. Pediatr. Nephrol..

[53] Team R.C. (2020). R: A Language and Environment for Statistical Computing. R Foundation for Statistical Computing.

[54] Frank E.H.J. (2020). rms: Regression Modeling Strategies, Volume R Package Version 6.1-0 ed..

[55] Violant-Holz V., Gallego-Jiménez M.G., González-González C.S., Muñoz-Violant S., Rodríguez M.J., Sansano-Nadal O., Guerra-Balic M. (2020). Psychological Health and Physical Activity Levels during the COVID-19 Pandemic: A Systematic Review. Int. J. Environ. Res. Public Health.

[56] Bauer L.L., Seiffer B., Deinhart C., Atrott B., Sudeck G., Hautzinger M., Rösel I., Wolf S. (2020). Associations of exercise and social support with mental health during quarantine and social-distancing measures during the COVID-19 pandemic: A cross-sectional survey in Germany. medRxiv.

[57] Schuch F.B., Bulzing R.A., Meyer J., Vancampfort D., Firth J., Stubbs B., Grabovac I., Willeit P., Tavares V.D.O., Calegaro V.C. (2020). Associations of moderate to vigorous physical activity and sedentary behavior with depressive and anxiety symptoms in self-isolating people during the COVID-19 pandemic: A cross-sectional survey in Brazil. Psychiatr. Res..

[58] Runacres A., Mackintosh K.A., Knight R.L., Sheeran L., Thatcher R., Shelley J., McNarry M.A. (2021). Impact of the COVID-19 Pandemic on Sedentary Time and Behaviour in Children and Adults: A Systematic Review and Meta-Analysis. Int. J. Environ. Res. Public Health.

[59] Reyes-Molina D., Alonso-Cabrera J., Nazar G., Parra-Rizo M.A., Zapata-Lamana R., Sanhueza-Campos C., Cigarroa I. (2022). Association between the Physical Activity Behavioral Profile and Sedentary Time with Subjective Well-Being and Mental Health in Chilean University Students during the COVID-19 Pandemic. Int. J. Environ. Res. Public Health.

[60] Okuyama J., Seto S., Fukuda Y., Funakoshi S., Amae S., Onobe J., Izumi S., Ito K., Imamura F. (2021). Mental Health and Physical Activity among Children and Adolescents during the COVID-19 Pandemic. Tohoku J. Exp. Med..

[61] Hallgren M., Nguyen T.T., Owen N., Stubbs B., Vancampfort D., Lundin A., Dunstan D., Bellocco R., Lagerros Y.T. (2020). Cross-sectional and prospective relationships of passive and mentally active sedentary behaviours and physical activity with depression. Br. J. Psychiatr..

[62] Jahrami H., BaHammam A.S., Bragazzi N.L., Saif Z., Faris M., Vitiello M.V. (2021). Sleep problems during the COVID-19 pandemic by population: A systematic review and meta-analysis. J. Clin. Sleep Med..

[63] Sharma M., Aggarwal S., Madaan P., Saini L., Bhutani M. (2021). Impact of COVID-19 pandemic on sleep in children and adolescents: A systematic review and meta-analysis. Sleep Med..

[64] Son C., Hegde S., Smith A., Wang X., Sasangohar F. (2020). Effects of COVID-19 on College Students’ Mental Health in the United States: Interview Survey Study. J. Med. Internet Res..

[65] Mulyadi M., Tonapa S.I., Luneto S., Lin W.T., Lee B.O. (2021). Prevalence of mental health problems and sleep disturbances in nursing students during the COVID-19 pandemic: A systematic review and meta-analysis. Nurse Educ. Pract..

[66] Killgore W.D.S., Taylor E.C., Cloonan S.A., Dailey N.S. (2020). Psychological resilience during the COVID-19 lockdown. Psychiatr. Res..

[67] Liu C.H., Zhang E., Wong G.T.F., Hyun S., Hahm H.C. (2020). Factors associated with depression, anxiety, and PTSD symptomatology during the COVID-19 pandemic: Clinical implications for U.S. young adult mental health. Psychiatr. Res..

[68] Shayganfard M., Mahdavi F., Haghighi M., Sadeghi Bahmani D., Brand S. (2020). Health anxiety predicts postponing or cancelling routine medical health care appointments among women in perinatal stage during the Covid-19 lockdown. Int. J. Environ. Res. Public Health.

[69] Wong S.Y.S., Zhang D., Sit R.W.S., Yip B.H.K., Chung RY-n Wong C.K.M., Chan D.C.C., Sun W., Kwok K.O., Mercer S.W. (2020). Impact of COVID-19 on loneliness, mental health, and health service utilisation: A prospective cohort study of older adults with multimorbidity in primary care. Br. J. Gen. Pract..

[70] Bélair M.A., Kohen D.E., Kingsbury M., Colman I. (2018). Relationship between leisure time physical activity, sedentary behaviour and symptoms of depression and anxiety: Evidence from a population-based sample of Canadian adolescents. BMJ Open.

[71] Mertens G., Gerritsen L., Duijndam S., Salemink E., Engelhard I.M. (2020). Fear of the coronavirus (COVID-19): Predictors in an online study conducted in March 2020. J. Anxiety Disord..

[72] Özdin S., Bayrak Özdin Ş. (2020). Levels and predictors of anxiety, depression and health anxiety during COVID-19 pandemic in Turkish society: The importance of gender. Int. J. Soc. Psychiatr..

