# Supplementary material for: The Association between Change in Lifestyle Behaviors and Mental Health Indicators in Immunosuppressed Individuals during the COVID-19 Pandemic
Source: Int J Environ Res Public Health. 2023 Jan 23;20(3):2099. doi: 10.3390/ijerph20032099 (PMC9916248; doi:10.3390/ijerph20032099)
Supplement: Supplementary file 1 [file ijerph-20-02099-s001.zip › ijerph-2125950-supplementary.pdf]

**Supplementary Table S1.** Continuous averages of lifestyle behaviors according to categorical measures [a].

| | MVPA [b] categories | | |
|---|---|---|---|
| | **Decrease (*n*= 43)** | **Stable (*n*= 63)** | **Increase (*n*= 26)** |
| **MVPA before COVID (days/wk)** | 5.0 ± 1.9 | 2.6 ± 3.1 | 3.3 ± 1.9 |
| **MVPA past 7 days (days/wk)** | 2.0 ± 1.8 | 3.2 ± 4.2 | 6.1 ± 2.3 |
| **MVPA Difference (days/wk)** | -3.0 ± 1.7 | 0.0 ± 0.0 | 2.4 ± 1.6 |
| **Sleep before COVID (min/day)** | 448.2 ± 61.1 | 465.2 ± 71.2 | 465.5 ± 75.7 |
| **Sleep past 7 days (min/day)** | 442.8 ± 103.5 | 465.5 ± 98.6 | 469.9 ± 77.3 |
| **Sleep Difference (min/day)** | -5.4 ± 86.1 | 0.3 ± 76.7 | 4.4 ± 78.7 |
| **Sedentary Behavior before COVID (min/day)** | 346.6 ± 242.5 | 379.5 ± 224.8 | 332.8 ± 231.8 |
| **Sedentary Behavior past 7 days (min/day)** | 497.6 ± 245.3 | 442.1 ± 208.6 | 425.0 ± 273.8 |
| **Sedentary Behavior Difference (min/day)** | 151.0 ± 142.5 | 62.6 ± 90.5 | 93.1 ± 145.0 |
| | **Sedentary categories** | | |
| | **Decrease (*n*= 12)** | **Stable (*n*= 30)** | **Increase (*n*= 90)** |
| **MVPA before COVID (days/wk)** | 3.7 ± 2.4 | 3.5 ± 2.8 | 3.9 ± 2.6 |
| **MVPA past 7 days (days/wk)** | 4.3 ± 3.7 | 3.3 ± 3.3 | 3.1 ± 3.3 |
| **MVPA Difference (days/wk)** | 0.3 ± 3.2 | -0.5 ± 1.20 | -1.1 ± 2.6 |
| **Sleep before COVID (min/day)** | 467.3 ± 55.8 | 432.9 ± 68.4 | 465.0 ± 67.9 |
| **Sleep past 7 days (min/day)** | 478.8 ± 115.2 | 428.1 ± 75.2 | 463.1 ± 100.1 |
| **Sleep Difference (min/day)** | 11.5 ± 102.9 | -4.8 ± 40.1 | -2.0 ± 87.9 |
| **Sedentary Behavior before COVID (min/day)** | 538.2 ± 217.1 | 442.6 ± 255.4 | 303.0 ± 207.2 |
| **Sedentary Behavior past 7 days (min/day)** | 449.3 ± 194.3 | 442.9 ± 255.2 | 471.4 ± 240.2 |
| **Sedentary Behavior Difference (min/day)** | -88.9 ± 61.7 | 0.3 ± 1.6 | 168.4 ± 110.4 |
| | **Sleep categories** | | |
| | Decrease (*n*= 44) | Stable (*n*= 37) | Increase (*n*= 51) |
| **MVPA before COVID (days/wk)** | 4.0 ± 2.6 | 3.5 ± 2.5 | 3.8 ± 2.8 |
| **MVPA past 7 days (days/wk)** | 3.3 ± 3.2 | 2.7 ± 2.5 | 3.7 ± 3.9 |
| **MVPA Difference (days/wk)** | -0.9 ± 2.9 | -0.8 ± 1.8 | -0.7 ± 2.5 |
| **Sleep before COVID (min/day)** | 468.5 ± 66.9 | 441.3 ± 59.6 | 460.8 ± 73.3 |
| **Sleep past 7 days (min/day)** | 378.4 ± 79.5 | 440.9 ± 59.6 | 535.3 ± 69.5 |
| **Sleep Difference (min/day)** | -90.1 ± 53.6 | -0.4 ± 1.7 | 74.5 ± 42.5 |
| **Sedentary Behavior before COVID (min/day)** | 329.0 ± 230.1 | 417.5 ± 277.1 | 335.0 ± 193.4 |
| **Sedentary Behavior past 7 days (min/day)** | 460.2 ± 271.5 | 490.8 ± 27.0 | 445.0 ± 172.3 |
| **Sedentary Behavior Difference (min/day)** | 131.2 ± 145.2 | 73.3 ± 126.3 | 110.0 ± 120.0 |

**Supplementary Table S2.** Associations between changes in lifestyle behaviors on mental health indicators in the subsample restricted to immunosuppressed individuals (*n* = 108).

| | **Stress** | | | **Distress** | | | **Resilience** | | | **Anxiety Symptoms** | | | **Depressive Symptoms** | | |
|---|---|---|---|---|---|---|---|---|---|---|---|---|---|---|---|
| | $\hat{\beta}$ | **95%CI** | *p* [a] | $\hat{\beta}$ | **95%CI** | *p* [a] | $\hat{\beta}$ | **95%CI** | *p* [a] | $\hat{\beta}$ | 95%CI | *p* [a] | $\hat{\beta}$ | 95%CI | *p* [a] |
| **MVPA** | | | | | | | | | | | | | | | |
| Stable (n=53) | ref | | | ref | | | ref | | | ref | | | ref | | |
| Increase (n=19) | -0.01 | ---0.47, 0.45 | 0.96 | 4.42 | -7.89, 16.73 | 0.48 | 1.62 | -1.14, 4.38 | 0.25 | 0.96 | -1.49, 3.42 | 0.44 | 0.63 | -2.15, 3.41 | 0.65 |
| Decrease (n=36) | 0.16 | - -0.21, 0.54 | 0.38 | **13.04** | **3.08, 22.98** | **0.01** | -0.72 | -2.96, 1.51 | 0.52 | **3.13** | **-1.15, 5.12** | **0.002** | **3.09** | **0.85, 5.34** | **0.007** |
| **Sedentary time** | | | | | | | | | | | | | | | |
| Stable (n=26) | ref | | | ref | | | ref | | | ref | | | ref | | |
| Increase (n=71) | **0.46** | **0.04, 0.89** | **0.03** | **15.59** | **4.21, 26.99** | **0.008** | -0.49 | -3.10, 2.12 | 0.71 | **2.43** | **0.09, 4.79** | **0.04** | **2.58** | **-0.05, 5.22** | **0.05** |
| Decrease (n=11) | **0.27** | **-0.35, 0.89** | **0.38** | **2.33** | **-14.36, 19.01** | **0.78** | -0.39 | -4.21, 3.44 | 0.84 | 0.48 | -2.96, 3.92 | 0.78 | 0.28 | -3.58, 4.14 | 0.88 |
| **Sleep duration** | | | | | | | | | | | | | | | |
| Stable (n=29) | ref | | | ref | | | ref | | | ref | | | ref | | |
| Increase (n=41) | **0.54** | **0.12, 0.96** | **0.01** | **17.58** | **6.09, 29.05** | **0.003** | -1.86 | -4.48, 0.73 | 0.16 | **2.53** | **0.16, 4.90** | **0.03** | **3.53** | **0.91, 6.15** | **0.008** |
| Decrease (n=38) | **0.62** | **0.19, 1.05** | **0.005** | **13.54** | **1.74, 25.35** | **0.02** | -0.62 | -3.30, 2.06 | 0.65 | **2.40** | **-0.03, 4.85** | **0.05** | **3.24** | **0.55, 5.93** | **0.02** |

[a] p value adjusted for age, sex, and physical illness diagnosis.
[b]MVPA = Moderate-to-vigorous physical activity.
**Bold** indicate statistically significant results at $p < 0.05$.
